# Towards efficient human–machine collaboration: effects of gaze-driven feedback and engagement on performance

**DOI:** 10.1186/s41235-018-0148-x

**Published:** 2018-12-29

**Authors:** Nikolina Mitev, Patrick Renner, Thies Pfeiffer, Maria Staudte

**Affiliations:** 1 CITEC, Universität des Saarlandes, Campus C7.4 (2.04), Saarbrücken, 66123 Germany; 2 CITEC, Bielefeld University, Inspiration 1, Bielefeld, 33619 Germany

**Keywords:** Human–computer interaction, Natural language generation, Listener gaze, Referential success, Multimodal systems

## Abstract

Referential success is crucial for collaborative task-solving in shared environments. In face-to-face interactions, humans, therefore, exploit speech, gesture, and gaze to identify a specific object. We investigate if and how the gaze behavior of a human interaction partner can be used by a gaze-aware assistance system to improve referential success. Specifically, our system describes objects in the real world to a human listener using on-the-fly speech generation. It continuously interprets listener gaze and implements alternative strategies to react to this implicit feedback. We used this system to investigate an optimal strategy for task performance: providing an unambiguous, longer instruction right from the beginning, or starting with a shorter, yet ambiguous instruction. Further, the system provides gaze-driven feedback, which could be either underspecified (“No, not that one!”) or contrastive (“Further left!”). As expected, our results show that ambiguous instructions followed by underspecified feedback are not beneficial for task performance, whereas contrastive feedback results in faster interactions. Interestingly, this approach even outperforms unambiguous instructions (manipulation between subjects). However, when the system alternates between underspecified and contrastive feedback to initially ambiguous descriptions in an interleaved manner (within subjects), task performance is similar for both approaches. This suggests that listeners engage more intensely with the system when they can expect it to be cooperative. This, rather than the actual informativity of the spoken feedback, may determine the efficiency of information uptake and performance.

## Significance statement

Can listener gaze facilitate goal-oriented human–machine collaboration? To solve a task jointly, interlocutors often need to establish a reference in a shared environment, e.g., to identify task-relevant objects. In such situated interactions, interlocutors typically use natural language, but other modalities, in particular gaze and gestures, support communicative success. In our work, we address the domain of assembly assistance and in particular object identification tasks. We show that an artificial speaker (a natural language generation (NLG) system) can improve task performance when providing gaze-aware proactive feedback based on a listener’s inspections of an object. In particular, giving information incrementally in subsequent chunks is more efficient than giving the description in one piece. Moreover, the feedback’s informativity not only leads to more efficient interactions but also influences the overall expectation for the capabilities of the NLG system. This expectation determines to what extent the listener wants to cooperate and will engage with the NLG system. The more intensely listeners engage with the system, the more effective is the information uptake and the better the task performance, even when some of the system’s responses are less informative.

## Introduction

In situated collaboration, spoken natural language is often used to refer to task-relevant objects in the form of *installments*. Installments are chunks of information uttered by a speaker to provide partial information to the listener in an incremental manner. Human speakers may produce installments without planning an entire unambiguous utterance. This effect is increased when they are under time pressure (Striegnitz et al. 2012). Using installments, speakers can quickly adapt to changes in the surroundings and in particular to the listeners’ feedback and actions. As shown by Zarrieß and Schlangen (2016), an artificial speaker can use installments to generate referring expressions effectively. This was considered intuitive and enhanced the identification of real objects depicted in static images.

In our research, we integrate listener feedback into the interaction loop by addressing the question of whether a listener’s gaze can successfully be used as a non-verbal feedback cue for adaptive installment generation. In particular, we investigate the interactions of an artificial speaker, i.e., a machine instructor, and a human listener. There is some evidence from studies in virtual environments that feedback from the artificial speaker based on listener gaze can increase interaction efficiency (Koller et al. 2012; Staudte et al. 2012; Garoufi et al. 2016). However, there are two remaining questions that we address in the present paper: (1) Can the successful use of listener gaze be replicated in real environments, which are much more complex to handle technically? (2) Can gaze-aware NLG be used to generate adaptive installments that provide references both incrementally and in the form of contrastive feedback? Specifically, we present a NLG system that monitors the gaze of the human listener and provides installments only if necessary, that is, if the listener’s gaze indicates wrong reference resolution. We further report on two experiments that evaluate the efficiency and the general perception of this behavior in comparison to long and exhaustive instructions. The results suggest that communication efficiency benefits from giving interactive and incremental instructions. However, in our experiments, this is preferred less by the users. Thus, there is a trade-off between efficiency and users’ preference in terms of perception.

Our approach draws on previous findings from (i) human–human interactions, which show that listener eye movements are closely tied, and time-aligned, to the current understanding of the comprehender and (ii) human–machine interactions, especially from work with assistive systems, in particular for assembly tasks, which show more generally that systems employing gaze as a communicative signal are socially beneficial—though sometimes less efficient. Below, we briefly review selected literature from those areas.

### Human–human interactions

To ensure communicative success in situated collaboration, speakers tend to observe listeners to detect if their communication message was received and understood correctly (Clark 1996). Listeners reliably inspect objects they believe are being referred to by the speaker (Tanenhaus et al. 1995; Eberhard et al. 1995). Consequently, speakers can monitor understanding and the mapping of meaning to the world by considering listener gaze (Clark and Krych 2004; Hanna and Brennan 2007; Brown-Schmidt 2012). Most of these studies, however, focus on the role of listener gaze as an index to the underlying comprehension processes. The benefit of gaze-based feedback cues for the speaker and successful reaction strategies are rarely examined. Human instruction givers might not be prepared to use technical cues based on listener gaze beneficially, as has been shown by Koleva et al. (2015). Coco et al. (2018), who examined the role of feedback and alignment in a “spot the difference task,” further found that their gaze aligned only if interlocutors could not exchange verbal feedback. Both results indicate that exploiting a technical augmentation of the listener gaze (e.g., by visualizing a gaze cursor) is not something that human speakers naturally do efficiently. In the studies described, the instructors were faced with the additional perception task of following gaze cursors, which might have increased the cognitive load too much. In contrast to this research, we focus on artificial speakers’ use of gaze feedback.

### Human–machine interactions

Gaze-based assistive systems have, along with the advances in mobile eye tracking technologies, moved into real-world environments in the last decade (Pfeiffer 2013). Our work is related to work in attentive assistance systems (Maglio et al. 2000) and human–robot and human–agent interactions, where gaze is relevant for the social aspects of interaction (Sidner et al. 2004) as well as for grounding verbal utterances using mechanisms of joint attention (Imai et al. 2003). Smart eyewear has been identified as a key technology for assistance systems (Pfeiffer et al. 2016a) and recently has been combined with a real-time analysis of eye tracking to support assembly tasks (Renner and Pfeiffer 2017; Blattgerste et al. 2017). Work on such assembly tasks has been done in both virtual and real worlds (Kopp et al. 2003; Kirk et al. 2007). However, projects including and examining the role of listener gaze are considerably less frequent. Fang et al. (2015) proposed a collaborative referring expression generation algorithm for situated human–robot interactions and used listener gaze to provide information incrementally. Their results surprisingly showed a performance drop when using listener gaze. This may, however, be explained by the method they used to interpret the gaze signal. We address this issue and apply the procedure for inspection detection proposed by Garoufi et al. (2016) to trigger verbal feedback that supplements instructions in our real-world assembly scenario.

### Contribution of this paper

We investigated the utility of listener gaze in a real-time object identification task. In particular, we designed, implemented, and employed an interactive NLG system using augmented reality technologies (Pfeiffer 2012; Pfeiffer and Renner 2014) to describe co-present objects to a human listener, who needs them for assembly. The system can monitor and react to listener gaze by generating verbal feedback to accept or reject the listener’s intentions as to which object to grasp next. We further compared two levels of instructional ambiguity and tested their effectiveness in two experiments: generating a long, unambiguous instruction vs. generating a short, ambiguous instruction followed by gaze-driven feedback. Furthermore, we examined the impact of feedback specificity by either providing an underspecified “No, not that one!” or contrastive feedback expressing the spatial relation of the target relative to the current gaze position (e.g., “Further left!”). We predicted that the interaction with the generation system would benefit from gaze-based feedback, despite the small-scale setting and the noise typically emerging in real-world and real-time interactions (in terms of movement and motion). We further hypothesized that this benefit might be large enough to compensate even for short, ambiguous instructions by the system and lead to similar if not shorter interaction times than for full exhaustive unambiguous instructions. Lastly, we predicted that contrastive feedback that provides additional referential information (i.e., after ambiguous instructions), incrementally and on demand, would be most efficient and lead to the shortest interaction times overall (cf. Zarrieß and Schlangen (2016) on installments and their efficiency).

## Experiment 1

**Fig. 1:**
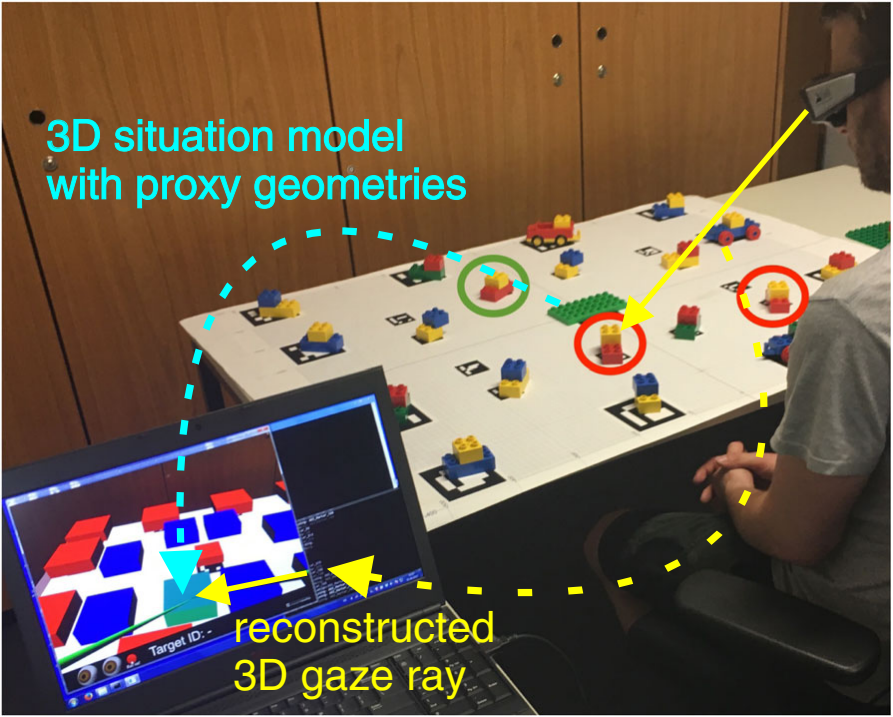
Initial setup. The listener is seated in front of the workspace before any objects have been collected. The picture shows a green circle around the target and red circles around competitors. The listener currently inspects the competitor object to the left as highlighted in the virtual 3D model. EyeSee3D is used to reconstruct the gaze ray in 3D (yellow). The target domain is modeled as a 3D situation model with boxes as proxies for the assembled structures (turquoise)

To investigate how listener gaze can be used in a dynamic task-oriented real-world interaction, we designed an assembly-like task and implemented a multimodal interactive system called GazInG (an interactive NLG system in a real environment), which can generate instructions in natural language. This system instructed a naive human listener to select and assemble building blocks. During the assembly process, the repeated identification of a specific object was required. By design, the scene was overloaded with many similar objects so that exhaustive object descriptions required naming two colors, sizes, and object types as well as locations. GazInG is further capable of monitoring and interpreting listener gaze and generating adaptive feedback. It relies on the EyeSee3D module, which models the environment as a 3D situation model using abstract geometry to represent the stimuli (see the turquoise arrow in Fig. 1).

In Experiment 1 we compared long but unambiguous instructions with ambiguous instructions that were followed by gaze-based feedback, which was either simple (underspecified feedback group) or contrastive (contrastive feedback group).

Two groups of participants completed the experiment. The underspecified feedback group received unambiguous instructions paired with no feedback on one block of trials, and ambiguous instructions paired with underspecified feedback on another block of trials. The contrastive feedback group received unambiguous instructions paired with contrastive feedback on one block of trials, and ambiguous instructions paired with contrastive feedback in another block of trials.

### Methods

#### Participants

Altogether, 48 participants, mainly students enrolled at Saarland University, took part in the experiment. Of these, 24 were assigned to each group: the underspecified feedback (19 female) and the contrastive feedback (16 female) groups. The average participant age of the underspecified feedback group was 25 years (19–35 years), and of the contrastive feedback group 24 years (20–31 years).

All participants were German native speakers and reported normal or corrected-to-normal vision and no red-green color blindness. Their participation was compensated for with €8 (underspecified feedback group) or €5 (contrastive feedback group) with the difference being due to the slightly shorter duration of the second group’s experiment.

#### Setup and apparatus

Figure 1 depicts our setup. We chose LEGO DUPLO as the target domain because the building blocks are of convenient, graspable size with easy to identify colors. At the same time, they offer a multitude of combinations and various ways of assembly. As the number and similarity of available objects in the workspace was high, it was not trivial to generate automatically unique identifying instructions (see Appendices A and B). A layout consisted of 20 composed objects with eight targets to be collected. Each composed object comprised two basic building blocks. The instructions did not provide guidelines on how to put together the selected elements but left this to the listener’s creativity. This was made clear in the task description. The participants had to build an individual LEGO model, based on the components the system instructed them to pick.

We used a binocular head-mounted eye tracker (SMI Eye Tracking Glasses) to collect gaze data. The tracker is equipped with a high-resolution scene camera (1280×960) recording at 24 Hz and two eye cameras recording at 30 Hz. The user’s head position and orientation are integrated by GazInG into a situation model in real time. This is realized by instrumenting the environment with low-cost printable fiducial markers (see the tablecloth in Fig. 1). These are located in known positions relative to the stimuli and are tracked by the scene camera of the eye tracker using computer vision. Fusing the thus-derived head position and orientation with eye tracking data from the glasses reconstructs the user’s gaze direction. This allows the system to cast a 3D gaze ray into the situation model (see the yellow arrow in Fig. 1). The intersections of the ray with the geometric models of the stimuli identify gazed-at objects. At this point, GazInG has semantically mapped the listener’s inspections. For further technical details of the approach, see Pfeiffer and Renner (2014) and Pfeiffer et al. (2016b). In this experiment, feedback was triggered by pooled inspections with a dwell time larger than 200 ms.

**Natural Language Generation** GazInG uses a heuristic approach to generate an instruction containing a referring expression that describes a composed object consisting of two basic building blocks on the fly given the domain knowledge. The syntactic structure of the instructions is predefined. The system is able to distribute the information needed to identify a target over several chunks. The first chunk, thus, realizes an ambiguous instruction, which can then be incrementally extended. Such an ambiguous instruction consists of a main clause that describes the bottom object. Its size and color are used as pre-modifiers and the head noun is randomly chosen from a set of synonyms for the type of object, as shown in Example (1). To output an unambiguous instruction, the algorithm appends two further post-modifiers: (i) a prepositional phrase or a relative clause to describe the top object and (ii) an adverbial phrase containing absolute position information (see Example (2)).

**Example (1)** Pick the big red building block.

**Example (2)** Pick the big red building block with the small yellow one on top at the back toward the left.

Inspections of target objects trigger positive feedback (e.g., “Yes”, “Exactly” etc.), and inspections of competitors trigger negative feedback signaling that the listener is considering a wrong object. This can be underspecified, e.g., “No, not that one!” or contrastive, providing relative position information, e.g., “Further left!” In the former case, the listener can exclude only the inspected competitor, which might be sufficient for simple scenes where fewer competitors are available in the visual context. In the latter case, however, the listener’s attention is directed towards the target from the relative gaze position. The system thereby reduces inspections of other competitors before the target is found and implements the notion of referring in installments, i.e., in chunks of information rather than one long referring expression.

#### Task

GazInG instructed a human listener to take a certain object; the listener performed grasping actions in response and assembled the LEGO objects in their own way. A total of eight objects had to be selected and taken from a single layout. Assembly continued with subsequent layouts. The final constructions were photographed and entered into a competition. The most creative result won a €10 Amazon voucher.

#### Procedure

We manipulated the *instructional ambiguity* within participants by presenting either unambiguous or ambiguous instructions to everyone. Further, we varied the gaze-driven verbal feedback and *feedback specificity* between groups. That is, the underspecified feedback group received unambiguous instructions without feedback and ambiguous instructions supplemented with underspecified feedback (extending on the design of Garoufi et al. 2016). On the other hand, the contrastive feedback group received contrastive feedback in both instructional ambiguity approaches. The feedback was more informative and meant to direct the listener’s attention toward the intended target, particularly after ambiguous instructions.

Participants were seated in front of the workspace and asked to listen carefully to and follow the system’s instructions. They were instructed to act as a team with the system and solve the task together as precisely as possible, i.e., to avoid taking the wrong building blocks. Then participants put on the eye tracking glasses and followed a three-point calibration procedure. Calibration was repeated between layouts and whenever needed. Before performing the actual task, a short practice session was completed: participants had to collect three targets among six objects in total to familiarize themselves with the task and the system’s pace.

The experiment consisted of two parts, one for each type of instructional ambiguity. In each part, the participants completed one layout, in which they searched for eight target objects. The order was balanced across participants. Each part consisted of working through one layout (see Appendix A). Participants were instructed to select an object as soon as they were sure which one was meant by the system. They heard a confirmation after a correct grasp action.

**Fig. 2:**
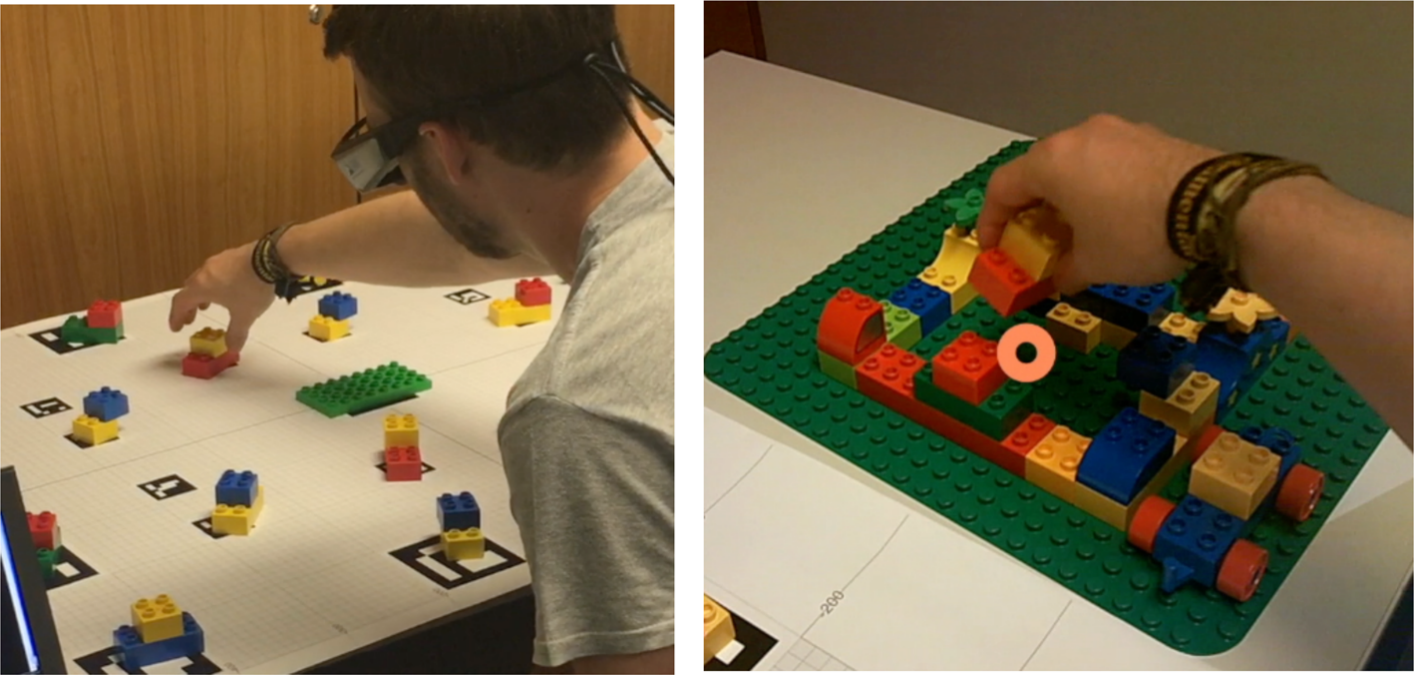
An example trial. The system instructs the user with an instruction possibly supplemented with verbal feedback. The listener identifies and grasps the target (left), which is assembled onto the other LEGO blocks (right). The circle represents the gaze cursor (not visible to the participant)

An example trial is presented in Fig. 2. In the following examples, the labels presented in brackets refer to the feedback specificity. Example (3) illustrates a typical interaction using unambiguous instructions for both groups.

**Example (3)**

SYSTEM: Pick the big red building block with a small yellow piece on top of it at the back toward the left.

LISTENER: [inspects the target]

SYSTEM: [silence]/ Yes, exactly! (underspecified/contrastive)

LISTENER: [grasps the target]

SYSTEM: Well done!

The underspecified feedback group experienced the ambiguous instructions usually as shown in Example (4).

**Example (4)**

SYSTEM: Pick the big red building block.

LISTENER: [inspects a competitor]

SYSTEM: No, not that one! (underspecified)

LISTENER: [inspects a competitor]

SYSTEM: No, not that one! (underspecified)

LISTENER: [inspects the target]

SYSTEM: Yes, exactly!

LISTENER: [grasps the target]

SYSTEM: Well done!

The contrastive feedback group may require fewer turns in the ambiguous instructions as shown in Example (5).

**Example (5)**

SYSTEM: Pick the big red building block.

LISTENER: [inspects a competitor]

SYSTEM: Further toward the left! (contrastive)

LISTENER: [inspects the target]

SYSTEM: Yes, that one!

LISTENER: [grasps the target]

SYSTEM: Well done!

After finishing a layout, participants filled in a questionnaire assessing their perception and impressions of their interaction with the system. Participants answered 13 questions to judge the interaction in each instructional ambiguity approach. Eight questions were followed by a five-point Likert scale (1 indicating a very good and 5 a poor score), e.g., “How good/precise did you find the spoken instructions?” or “How flexible did you find the interaction?” In addition, there were five yes/no questions, such as “Was the system’s feedback confusing?” to assess if the interaction with the system felt natural. The question “Were the instructions exhaustive, i.e., you were able to identify a target upon hearing the instruction?” checked whether the participants paid attention. In a final questionnaire, they were asked five yes/no questions to compare both interaction strategies and assess user preferences. The experiment lasted between 30 and 45 minutes.

#### Analyses

**Fig. 3:**
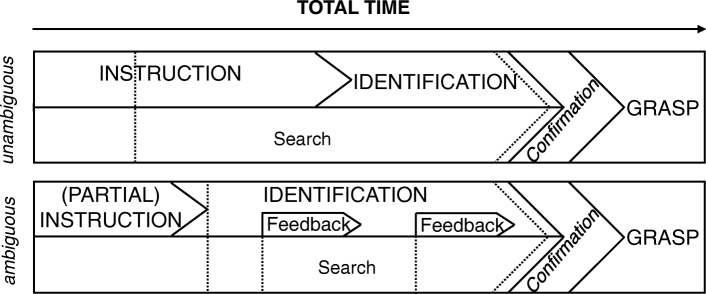
Interaction phases for both strategies. The spoken instruction, followed by identification, i.e., time to first target inspection, and the grasp of the object after a verbal confirmation is given. The visual search starts either during or after instruction and can be interleaved with feedback, depending on the approach

All measures were collected on a per-item basis. Performance was measured using the total time from instruction onset until a target was grasped, and whether the interaction ended successfully with the correct object selected. The total time was further divided into three phases, which differ depending on the instructional ambiguity (Fig. 3). The first phase is determined by the duration of the spoken instruction, from speech onset to speech offset. Secondly, we assessed identification, the time needed from the offset of the instruction to the listener’s first inspection of the target. Finally, the time from the first target inspection until the grasp of the target determined the duration of the third phase.

We further counted the number of feedback occurrences per interaction and also assessed the time from instruction offset to the first positive or first negative feedback instance, which marks the end of the (initial) visual search for the target.

Statistical analyses were conducted in the R statistical programming environment (R Core Team 2014). We assessed statistical significance using linear mixed-effects models using the lme4 package in R and model comparison to determine the influence of instructional ambiguity and feedback specificity. As proposed by Bates et al. (2015), we started with the maximal model fitting our assumptions with respect to the random effects structure. If the models failed to converge, we simplified the random structure by first removing the correlations between random slopes and intercepts, followed by the intercept terms, starting with the random effect for items (if present).

### Results

The results reported in this section are based on 722 unique trials after outliers had been removed (data points that are 2.5 standard deviations above or below the mean) from a total of 768.

#### Total time

The time to solve each task, i.e., to find and collect a building block, indicates the degree of efficiency of the communication with the system. All tasks were solved, and there were only a few wrong grasps (8.7%), as well as almost no need for repetition of an instruction, showing that both interaction strategies are effective. Table 1 summarizes the response times for the interaction phases. Specifically, the underspecified feedback group was faster at solving the task after listening to an unambiguous instruction (*M*=14.31 s, standard deviation SD =8.60 s) than to an ambiguous instruction with underspecified feedback (*M*=17.56 s, SD =10.44 s). For the contrastive feedback group, the direction of the effect changed. The ambiguous instruction now led to shorter task completion time (*M*=11.96 s, SD =5.61 s) compared to following the unambiguous instruction (*M*=12.75 s, SD =4.75 s). Specifically, we constructed an individual model for each group with instructional ambiguity as a fixed effect and with random intercepts and slopes for subjects and items. Both comparisons revealed the main effects of instructional ambiguity: for the underspecified feedback group, *χ*^2^(1)=4.008 with *p*<0.05, and for the contrastive feedback group, *χ*^2^(1)=4.502 with *p*<0.05. For the subset of ambiguous instructions in both groups, we fitted a linear mixed-effects model with feedback specificity as a fixed effect and included random intercepts and slopes for subjects and items. There was a main effect of feedback specificity on total time revealed by model comparison (*χ*^2^(1)=15.907,*p*<0.001), that is, contrastive feedback improved task completion time over underspecified feedback.

**Table 1:** Mean durations of the interaction phases in Experiment 1

Instructional ambiguity	Feedback specificity	Instruction	Identification	Grasp	Total time
		[s]	[s]	[s]	[s]
Unambiguous	Absent	7.21	2.17	4.93	14.31
Ambiguous	Underspecified	2.81	7.22	7.52	17.56
Uunambiguous	Contrastive	7.23	1.27	4.25	12.75
Ambiguous	Contrastive	2.80	4.21	4.94	11.96

The first two rows refer to the underspecified feedback group and the last two rows refer to the contrastive feedback group

#### Identification time

Next, we analyzed the time needed to find and inspect the intended target after instruction offset. Unsurprisingly, participants were quicker at identifying a target following an unambiguous instruction, as it contains all the information needed. In addition, they could start searching as soon as they had heard the first part of the instruction. Analogously to the analysis of the total time, we fitted linear mixed-effects models for each data set with the same random structure. Model selection revealed two main effects for our within-subject manipulation of instructional ambiguity: for the underspecified feedback group, *χ*^2^(1)=60.257 with *p*<0.001, and for the contrastive feedback group, *χ*^2^(1)=92.868 with *p*<0.001. Additionally, we analyzed our between-subject manipulation and observed a main effect of feedback specificity for the ambiguous approach (*χ*^2^(1)=4.172,*p*<0.05). In other words, listeners needed three times longer after hearing an ambiguous instruction (*M*=7.22 s, SD =8.37 s) to find the target object than after listening to an unambiguous one (*M*=2.17 s, SD =5.12 s). This time was shortened dramatically when gaze-driven contrastive feedback followed the instructions, though listeners still inspected the intended target sooner after the unambiguous instructions (*M*=1.27 s, SD =2.21 s) than after the ambiguous instruction (*M*=4.21 s, SD =3.80 s).

#### Feedback occurrences

We analyzed the number of negative feedback instances that occurred after the ambiguous instructions across groups, but surprisingly, there was no significant difference (*p*=0.658).

**Fig. 4:**
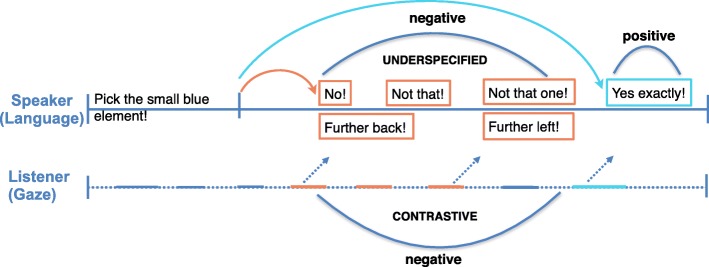
Typical trial and the differentiation of feedback specificity. The red arrows represent the durations considered in the feedback analysis

We further examined how much time elapsed until a feedback instance was triggered through listener gaze after the ambiguous instructions, in both groups. Specifically, we contrasted this time for the first negative with the first positive feedback instance in an interaction (see red arrows in Fig. 4), since this indexes the visual search and how actively and intensively participants engaged with the instruction-giving system.

**Fig. 5:**
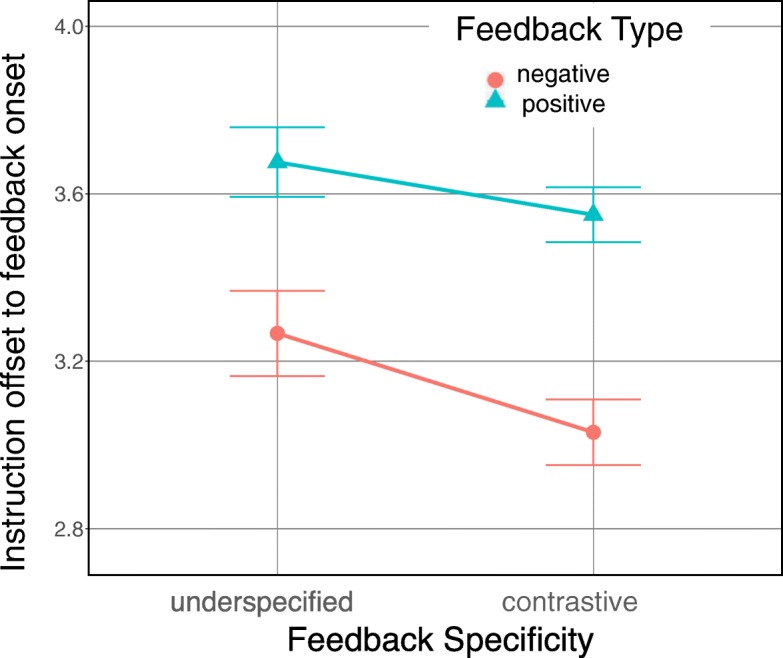
Time interval from the instruction offset to the onsets of the first negative (triggered by a competitor inspection) and first positive (triggered by a target inspection) feedback instances for the ambiguous instructions in Experiment 1 (log transformed with 95% confidence interval error bars)

Figure 5 depicts the respective means. For the analysis, we fitted a model with feedback specificity as a fixed effect and with random intercepts and slopes for subjects and items. Importantly, we found a main effect of feedback specificity (*χ*^2^(1)=18.416,*p*<0.001). As expected, the pattern observed for the identification time (determined by first target inspection) persists for the time to first positive feedback instance because it is precisely this inspection that triggers the first positive feedback instance. The underspecified feedback group provoked positive feedback later (*M*=10.33 s, SD =16.91 s) than the contrastive feedback group (*M*=5.43 s, SD =5.97 s). This demonstrates how more specific feedback narrowed down the search for the target object and shortened the time to find it. Furthermore, the investigation of the first occurrence of a negative feedback instance revealed that listeners also inspected a competitor matching the description faster after the contrastive feedback (*M*=1.97 s, SD =2.68 s) than after the underspecified (*M*=4.07 s, SD =5.77 s) feedback. This suggests that listeners’ expectation of an informative response elicits more deliberate and controlled use of gaze to engage better with the system because it constantly responds to it with useful information restricting the search space. They could use their gaze feedback to probe actively.

#### Questionnaires

**Fig. 6:**
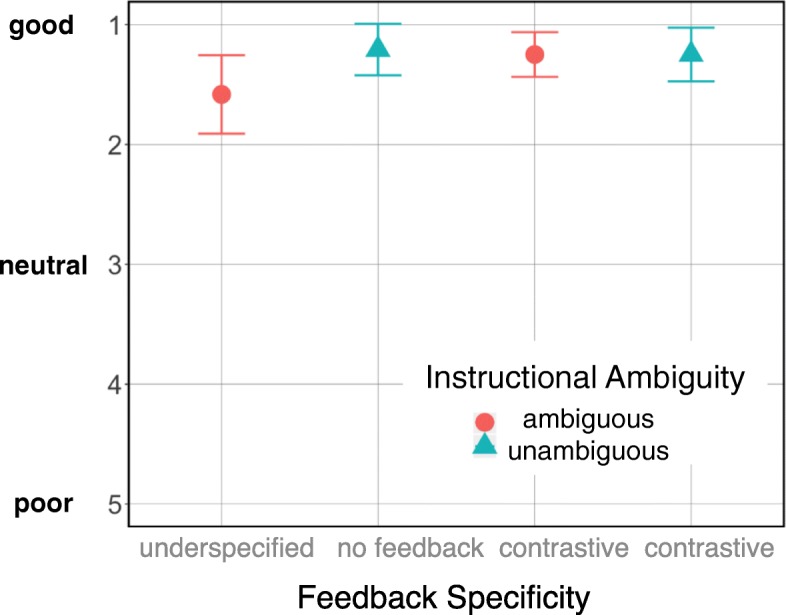
Mean responses for “How good did you find the interaction flow?” for Experiment 1 on a five-point Likert scale, where 1 indicates a good/positive rating and 5 a poor one. (95% confidence interval error bars)

Overall the interaction with the system was perceived as rather natural and the gaze-driven feedback was rated as helpful and not confusing. Interestingly, there was a clear preference in both groups for listening to and following an unambiguous instruction. All participants (100%) in the underspecified feedback group and most of the participants in the contrastive feedback group (87.5%) stated that they preferred unambiguous instructions and indicated them as more pleasant, although the contrastive feedback group was faster when responding to ambiguous instructions. We ran a simple linear regression on the responses of each group to the question “How good did you find the interaction flow?” (Fig. 6) and observed a marginal effect of instructional ambiguity for the underspecified feedback group (*β*=−0.375,*t*(46)=−1.98,*p*=0.0537). Further, for the subset of ambiguous instructions, simple linear regression revealed an effect of feedback specificity approaching significance (*β*=−0.333,*t*(46)=−1.829,*p*=0.0739). That is, when contrastive feedback followed an ambiguous instruction, it was judged to be better (*M*=1.25, SD =0.44) than when underspecified feedback was provided (*M*=1.58, SD =0.77). The former assessment was like the perception of the unambiguous instructions by the contrastive feedback group (*M*=1.25, SD =0.53) and the underspecified feedback group (*M*=1.20, SD =0.51). This, and similar results from the other questions, demonstrates that the informativity of the verbal feedback mitigates for the instructional ambiguity when giving initially partial, ambiguous instructions and so listeners experience it as smoother.

### Discussion

Our data provide some evidence for the successful use of listener gaze in a real-world task. Instructional ambiguity that refers to objects incrementally and reacts to listeners’ gaze can be used to identify objects in the shared space. Moreover, the performance results indicate that feedback specificity is essential for efficiency. The results reveal that contrastive feedback benefits task performance because it not only warns the listener against grasping a wrong object, but also includes a relative direction in which to look further for the target. In contrast, underspecified feedback merely prevents the user from wrong grasps, and does not facilitate the search. Notably, the combination of ambiguous instructions with contrastive feedback numerically even outperformed unambiguous instructions.

Interestingly, there was a mismatch in the perception and performance measures with respect to unambiguous and ambiguous instructions. Apparently, listeners felt more confident in their own performance when following unambiguous instructions. One reason for this might be that the unambiguous instructions allowed participants to remain rather passive until the grasp action. After an ambiguous instruction, in contrast, they had to engage actively with the system to make progress in the task. The former is considered as more convenient despite being apparently less efficient compared to the more interactive strategy, i.e., ambiguous instructions with specific contrastive feedback. Whether this behavior emerges as a direct response to the system’s behavior in a given trial or whether this is a result of a more global adaptation to the system was investigated in Experiment 2.

## Experiment 2

By giving only ambiguous instructions, this experiment further examined the impact of feedback specificity on task performance. Feedback specificity was manipulated within participants and in an interleaved and randomized order, item by item. Thus, participants did not know in advance which type of feedback they might receive. This aimed at assessing whether participants benefited from the contrastive feedback in the first experiment because more information was conveyed, so that this system is inherently more efficient—or whether more generally the participants adapted to the system, e.g., by increasing their attendance or willingness to collaborate and thus, to really take up and process the information provided efficiently. If the former hypothesis holds, then performance with contrastive feedback would remain high (and higher than with underspecified feedback), even if interleaved. If the latter hypothesis is true, we would expect to see either low performance in both approaches (since engagement decreases altogether) or high performance in both approaches (since engagement is high and leads to more efficient information uptake).

### Participants

Altogether, 24 German native speakers (16 female) participated in the experiment. We made sure none of them had already participated in Experiment 1. The average age of the participants was 24 years (18–32 years). They reported normal or corrected-to-normal vision and no red-green color blindness, and were compensated with €7.

### Procedure

The task was the same as in Experiment 1 and the procedure was almost identical. This time, the experiment consisted of four parts to produce a sufficient amount of data per approach, and so two more layouts were designed (see Appendix). The order was balanced across approach. In contrast to the procedure in Experiment 1, there was no questionnaire, but after finishing all four parts, the participants answered two questions: whether they noticed any differences and whether they had a particular strategy for inspecting objects. The experiment lasted around 40 minutes.

### Results

The total number of observations was 768 and 737 remained after outliers had been removed (data points that are 2.5 standard deviations above or below the mean). Table 2 summarizes the response times for the three trial phases (Fig. 3).

**Table 2:** Mean durations of the three interaction phases and the total time for Experiment 2

Feedback specificity	Instruction	Identification	Grasp	Total time
	[s]	[s]	[s]	[s]
Underspecified	2.81	4.84	4.98	12.63
Contrastive	2.79	4.47	5.07	12.33

#### Total time

**Fig. 7:**
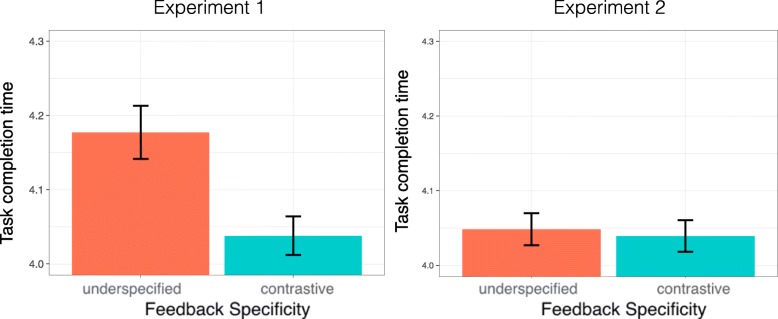
Total time for the interactions for Experiment 1 with ambiguous instructions (left) and for Experiment 2 (right)

Figure 7 compares the total time needed to finish the task for ambiguous instructions in both experiments. In contrast to the data for the subset of ambiguous instructions from Experiment 1 (left), there was no significant difference in performance for the two approaches in Experiment 2 (right). When participants received underspecified feedback, the task completion time was slightly longer (*M*=12.63 s, SD =6.83 s) than following contrastive feedback (*M*=12.33 s, SD =6.52 s). We fitted a model with feedback specificity as a fixed effect and with random intercepts and slopes for subjects and items but there was no significant effect of feedback specificity (*χ*^2^(1)=0.666,*p*=0.414).

#### Identification time

We fitted a model with feedback specificity as a fixed effect but, unlike Experiment 1, we did not find a significant difference between the two approaches in Experiment 2 (*χ*^2^(1)=0.065,*p*=0.799). In general, listeners in this experiment were a bit slower to inspect the target compared to those receiving contrastive feedback in Experiment 1, though they considered the target sooner when feedback was contrastive (*M*=4.47 s, SD =5.32 s) than when it was underspecified (*M*=4.84 s, SD =4.82 s).

#### Feedback occurrences

**Fig. 8:**
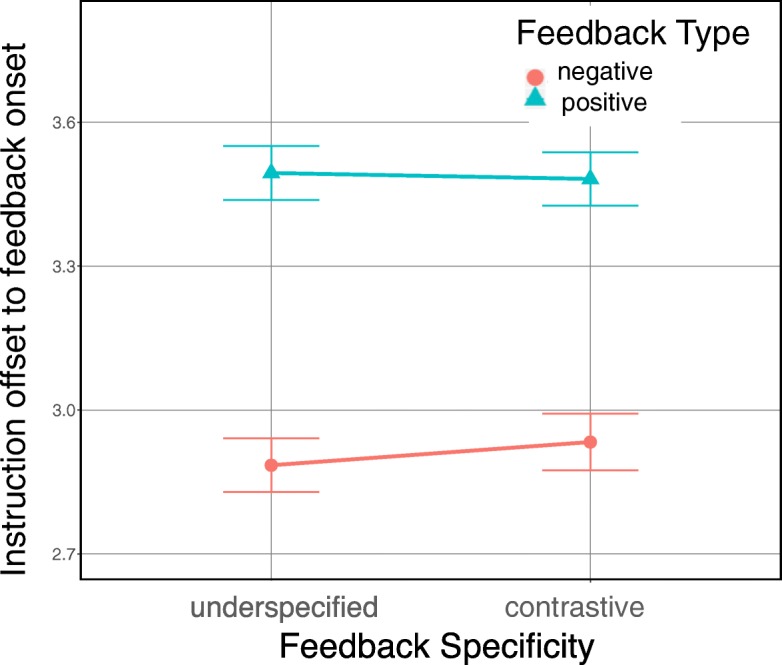
Time interval from instruction offset to the onsets of the first negative and first positive feedback instances in Experiment 2

The type of instructions (instructional ambiguity) was not manipulated in this experiment, i.e., the system systematically generated ambiguous instructions. However, verbal feedback can be considered as a dependent variable, since it is a direct consequence of participants’ visual search behavior. Competitor inspections triggered negative feedback and target inspections triggered positive feedback. It is the negative feedback that differs in specificity. As was done for Experiment 1, we constructed a generalized linear mixed-effects model (with a logit link function) fitted to feedback occurrences with feedback specificity as a fixed effect. There was a significant effect of feedback specificity on the number of feedback occurrences (*β*=0.168, standard error of the mean SE=0.070,*z*=2.396,*p*=.017). That is, when listeners followed underspecified feedback, their gaze triggered more negative instances (*M*=2.19 instances, SD =1.56 instances), i.e., they considered more competitors before arriving at the target, in comparison to when they followed contrastive feedback (*M*=1.74 instances, SD =1.10 instances). Furthermore, we ran a sequential analysis on feedback occurrences to assess the first relevant inspections using a linear mixed-effects model with feedback specificity as a fixed effect. Unlike Experiment 1, there is no significant difference with respect to our manipulation of feedback specificity (*χ*^2^(1)=0.100,*p*=0.752). Interestingly, listeners quickly inspected a competitor object (Fig. 8) that triggered the first negative inspection and this happened comparably soon in both types of feedback (underspecified, *M*=1.45 s, SD =2.19 s; contrastive, *M*=1.79 s, SD =3.34 s).

### Discussion

The results suggest that the chance to receive more informative feedback influences the overall willingness to interact and cooperate with a system on solving a task. Listeners seem to have greater expectations for the capabilities of the system, which is reflected in their gaze behavior. Thus, participants in the more difficult and rather unnatural approach (ambiguous instructions with underspecified feedback) were now as efficient as those experiencing the more informative approach (ambiguous instructions with contrastive feedback). These findings provide some evidence that participants can deal effectively with the imperfect behavior of a system if they perceive it as helpful and efficient overall. In other words, it is not solely the actual informativity of the spoken output in a given trial, but the confidence in the system’s supportive behavior more generally, that determines how efficient information uptake is. In terms of the strategic use of gaze, it seems as if none of the participants spotted the manipulation. This became evident from the short written answers they gave to the question “Did you notice any specific difference across the experiment?” after completing the online experiment. The differences some participants mentioned concerned the visual scenes and not the system’s verbal output. Specifically, participants first looked at everything but relied on the system’s instructions to find a target. Thus, we assume that listeners adapted their engagement and behavior rather naturally and unconsciously instead of employing a tactic for where to look and which object to inspect next in a specific experimental approach.

## General discussion

Interactive systems that use natural language in situ to assist a user in solving a task can benefit from exploiting listener gaze. Although the gaze signal is continuous and rapid, Koller et al. (2012) showed that it can be exploited effectively by a NLG system designed to give directions to a listener and to refer to objects in a virtual environment. In their study, they showed that using listener gaze led to higher success rates. Real-world interactions are noisier and the system’s knowledge about the environment is usually far from perfect. Thus, it is more challenging to make use of listeners’ eye movements in such a setting. Further, Koleva et al. (2015) report that even human speakers did not benefit from seeing where the listeners were looking when following the speakers’ spontaneous instructions for selecting certain objects. Perhaps it is difficult for a human speaker constantly to monitor and interpret the gaze signal. Alternatively, the mediation of gaze information by a gaze pointer overlaid on a scene camera video, as used in that study, created an artificial situation that speakers could not exploit intuitively and efficiently.

In contrast, we employed an artificial speaker, that is, a parametrized NLG system, which tracks users’ eye gaze to real objects while simultaneously planning an utterance. This system has the advantage of generating instructions systematically and without the great variation that is typical of instructions from humans. Such control over the (artificial) speaker allows us to integrate different modalities in the interaction without much additional effort while avoiding recursive effects between independent and dependent variables (a variation by the speaker would affect listener behavior, which in turn could affect the speaker). Lastly, providing gaze-driven feedback triggered by object inspections is computationally inexpensive for our system but enables it to be even more interactive and to engage better with the listener.

The results from our experimental investigations with this system support this view and suggest that exploiting listener gaze in real-world human–machine collaborations can indeed be beneficial. Our results extend previous research by looking at interactions with increased interactivity with an assistance system. Instead of generating long unambiguous instructions with all the required information, our system splits the information and provides it on demand, by giving partial instructions and requiring a non-verbal cue from the listener to progress the communication. While Experiment 1 showed that this might be considered more demanding, even exhausting, as listeners were more involved, the assessment of using such variants of installments to refer to co-present objects (i.e., ambiguous instruction with contrastive feedback) revealed that the interaction flow was perceived positively and rated as highly as following an unambiguous instruction. Moreover, instructional ambiguity that refers to objects incrementally and reacts to listeners’ gaze can be used to identify objects in the shared space more quickly.

Experiment 2 then examined whether the benefit of contrastive feedback is inherent to it or whether there is a learning effect specific to this system’s behavior. Here, the system provided underspecified or contrastive feedback in an interleaved manner. Somewhat surprisingly, the results revealed that both approaches now led to equally high task performance. Participants were equally efficient in completing the task when listening to underspecified or contrastive feedback given the different study designs; however, obtaining different results is not that unusual (Charness et al. 2012). Specifically, we interpret the performance gain in Experiment 2 as a natural adaptation to the system’s informative behavior that extends to and even absorbs the not-so-informative trials. Supportive evidence for this interpretation comes from the sequential feedback analysis, which shows that gaze was used more deliberately, and this helps participants to advance quickly within a trial.

Lastly, given that not only does the specificity of gaze-driven feedback improve task performance, but the listener’s perception of an assistive system also influences it, changing the form of the instructions could possibly contribute further to efficiency. A direction for future research could be to vary the syntactic structure and the lexicalization when generating an ambiguous instruction to examine the effect of politeness in this context (Pemberton 2011).

## Conclusion

In this paper, we extend previous findings from virtual environments and show that underspecified feedback (e.g., “No, not that one!”) combined with ambiguous instructions cannot compete, in terms of interaction efficiency, with unambiguous instructions even when these are not followed by (confirmatory) feedback. Combining an ambiguous instruction with contrastive feedback, however, increased efficiency dramatically and this approach even outperformed unambiguous instructions (Experiment 1). Moreover, we provide evidence that contrastive feedback determines the overall strategy for efficient information uptake by the human listener, even if the system’s output varies in informativity (or rather feedback specificity, as in Experiment 2). The expectation of obtaining another piece of information in response to their own behavior seems to influence the listener’s readiness to team up with the system to solve a task, and thus, to employ their eye gaze intensively.

In summary, we have shown that listener gaze is a reliable cue for indicating reference resolution and that exploiting it to give contrastive feedback with a position specification (e.g., “Further left!”) relative to the listener’s current gaze point drastically improves performance in task-oriented real-world human–machine interactions.

## Appendix A: Layouts 1 and 2 used in Experiments 1 and 2

**Fig. 9:**
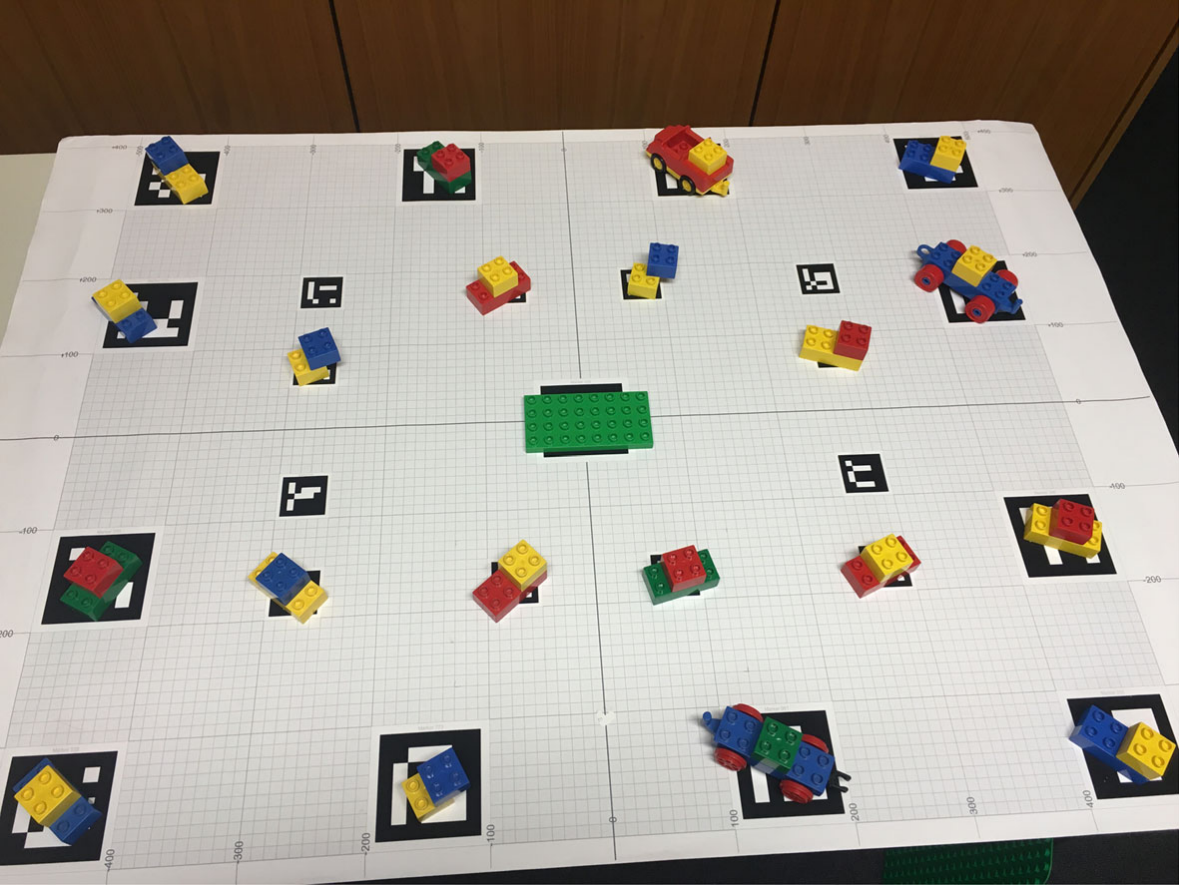
First scene layout

**Fig. 10:**
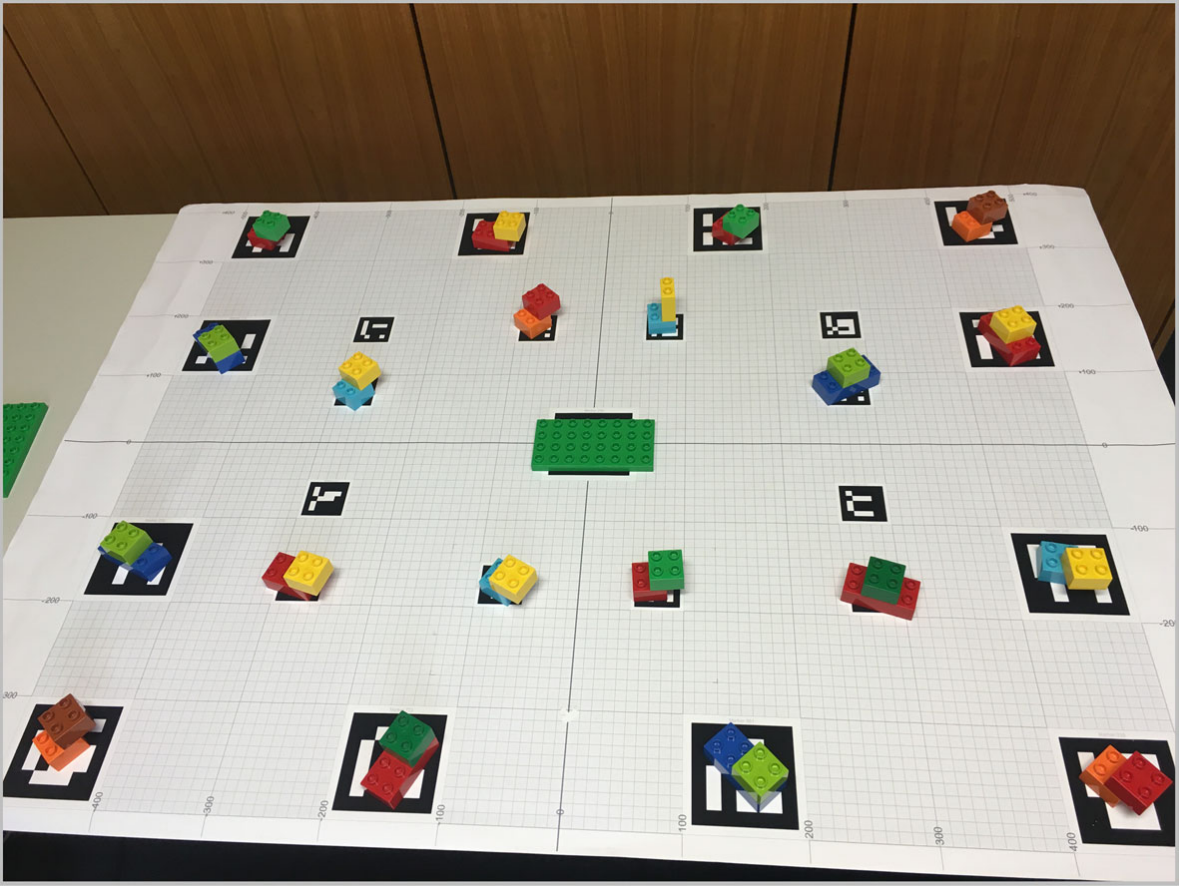
Second scene layout

## Appendix A: Layouts 3 and 4 used in Experiment 2

**Fig. 11:**
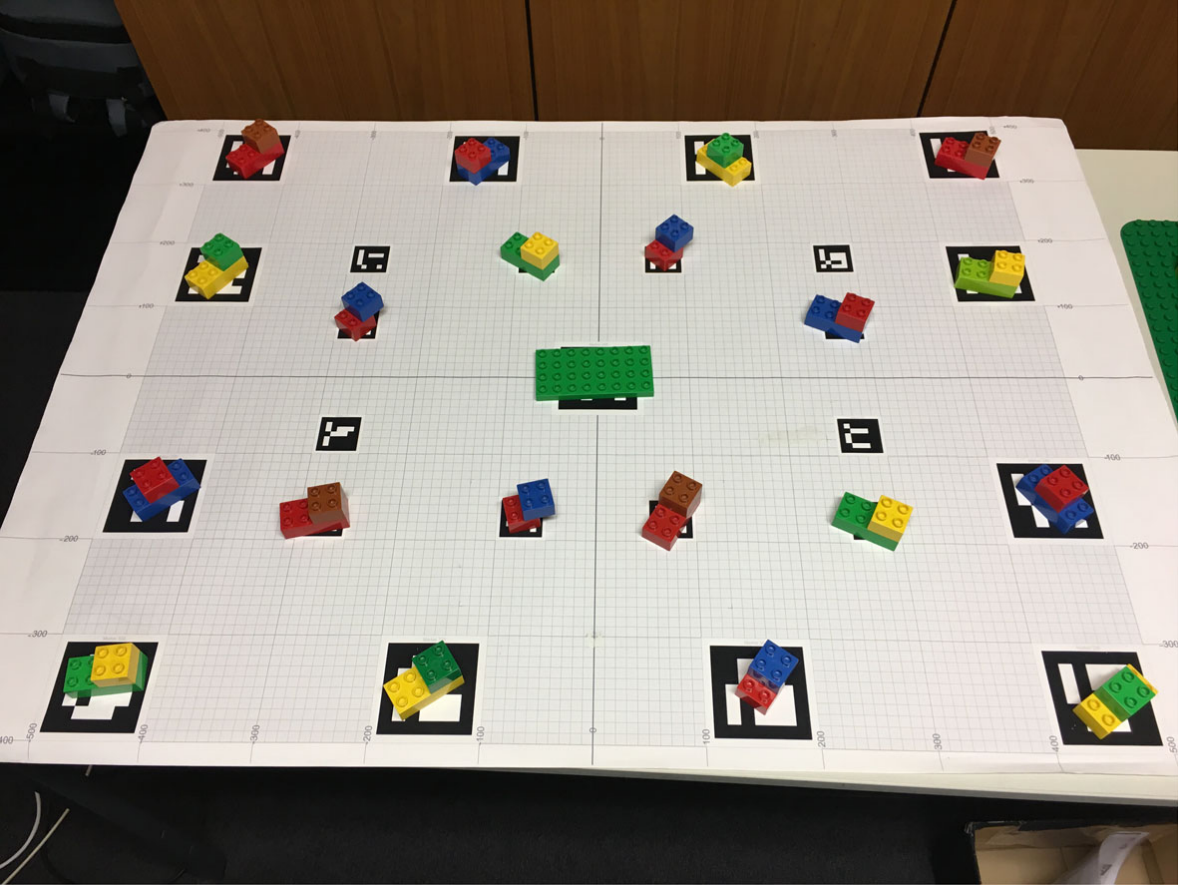
Third scene layout

**Fig. 12:**
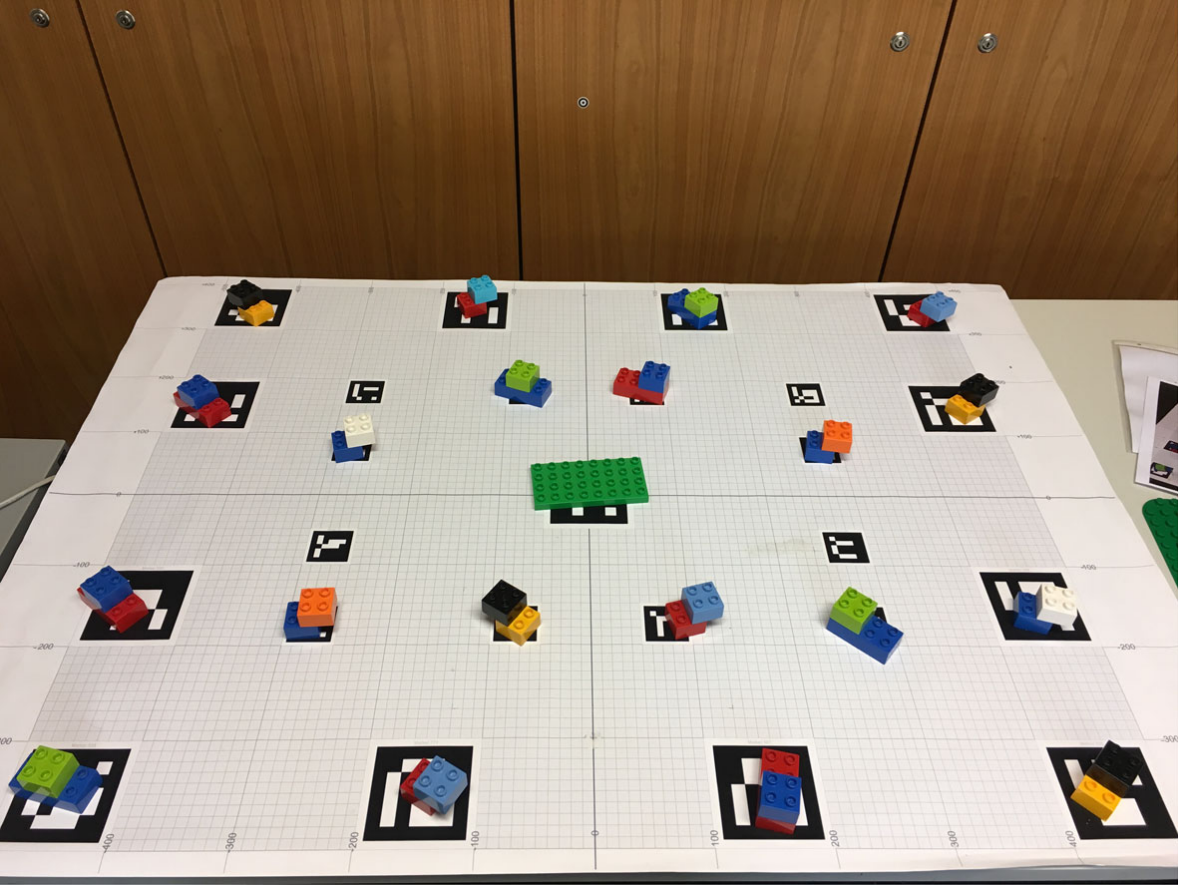
Fourth scene layout

## Abbreviations

NLG: Natural language generation
SD: Standard deviation

## References

[CR1] Bates, D., Kliegl, R., Vasishth, S., & Baayen, H. (2015). *Parsimonious mixed models* Vol. 1506. http://arxiv.org/abs/1506.04967.

[CR2] Blattgerste, J., Strenge, B., Renner, P., Pfeiffer, T., & Essig, K. (2017). Comparing conventional and augmented reality instructions for manual assembly tasks. In *Proceedings of the 10th International Conference on Pervasive Technologies Related to Assistive Environments*. 10.1145/3056540.3056547 (pp. 75–82). ACM.

[CR3] Breazeal, C., Kidd, C. D., Thomaz, A. L., Hoffman, G., & Berlin, M. (2005). Effects of nonverbal communication on efficiency and robustness in human–robot teamwork. In *2005 IEEE/RSJ International Conference on Intelligent Robots and Systems*. 10.1109/IROS.2005.1545011 (pp. 708–713).

[CR4] Brown-Schmidt S. (2012). Beyond common and privileged: Gradient representations of common ground in real-time language use. Lang Cogn Process.

[CR5] Charness G., Gneezy U., Kuhn M. A (2012). Experimental methods: Between-subject and within-subject design. Journal of Economic Behavior & Organization.

[CR6] Clark H. H. (1996). Using language. Cambridge: Cambridge University Press, Pp. xi 432. J Linguist.

[CR7] Clark, H. H.,& Krych, M. A (2004). Speaking while monitoring addressees for understanding. *Journal of Memory and Language*, *50*(1), 62–81. 10.1016/j.jml.2003.08.004.

[CR8] Coco M. I., Dale R., Keller F. (2018). Performance in a collaborative search task: The role of feedback and alignment. topiCS.

[CR9] Eberhard K. M., Spivey-Knowlton M. J., Sedivy J. C., Tanenhaus M. K (1995). Eye movements as a window into real-time spoken language comprehension in natural contexts. Journal of Psycholinguistic Research.

[CR10] Fang R., Doering M., Chai J. Y. (2015). Embodied collaborative referring expression generation in situated human–robot interaction. Proceedings of the 10th Annual ACM/IEEE International Conference on Human–Robot Interaction, HRI ’15.

[CR11] Fischer K. (2015). The Effects of Social Gaze in Human-Robot Collaborative Assembly. Social Robotics. ICSR 2015. Lecture Notes in Computer Science, vol 9388.

[CR12] Garoufi K., Staudte M., Koller A., Crocker M. W. (2016). Exploiting listener gaze to improve situated communication in dynamic virtual environments. Cognitive Science.

[CR13] Hanna J. E., Brennan S. E. (2007). Speakers’ eye gaze disambiguates referring expressions early during face-to-face conversation. Journal of Memory and Language.

[CR14] Imai M., Ono T., Ishiguro H. (2003). Physical relation and expression: Joint attention for human–robot interaction. IEEE Transactions on Industrial Electronics.

[CR15] Kirk D., Rodden T., Fraser D. S. (2007). Turn it this way: Grounding collaborative action with remote gestures. Proceedings of the SIGCHI Conference on Human Factors in Computing Systems, CHI ’07.

[CR16] Koleva, N., Hoppe, S., Moniri, M. M., Staudte, M., & Bulling, A. (2015). On the interplay between spontaneous spoken instructions and human visual behaviour in an indoor guidance task. In *Proceedings of the 37th Annual Meeting of the Cognitive Science Society, CogSci 2015*. July 22-25, 2015, https://mindmodeling.org/cogsci2015/papers/0204/index.html. Pasadena, California, USA.

[CR17] Koller A., Staudte M., Garoufi K., Crocker M. (2012). Enhancing referential success by tracking hearer gaze.

[CR18] Kopp S., Jung B., Leßmann N., Wachsmuth I (2003). Max—A multimodal assistant in virtual reality construction. KI.

[CR19] Maglio P. P., Matlock T., Campbell C. S., Zhai S., Smith B. A (2000). Gaze and speech in attentive user interfaces. Proceedings of the Third International Conference on Advances in Multimodal Interfaces, ICMI ’00.

[CR20] Pemberton L. (2011). Politeness in interaction design. Romanian Journal of Human-Computer Interaction.

[CR21] Pfeiffer, T. (2012). Using virtual reality technology in linguistic research. In Coquillart S., Feiner S., Kiyokawa K. (Eds.) In *Virtual Reality Short Papers and Posters (VRW), Institute of Electrical and Electronics Engineers (IEEE)*. 10.1109/VR.2012.6180893 (pp. 83–84).

[CR22] Pfeiffer, T. (2013). *Gaze-based assistive technologies*, (pp. 90–109). IGI Global. 10.4018/978-1-4666-4438-0.ch004.

[CR23] Pfeiffer T., Renner P. (2014). EyeSee3D: a low-cost approach for analyzing mobile 3D eye tracking data using computer vision and augmented reality technology. Proceedings of the Symposium on Eye Tracking Research and Applications (ETRA ’14).

[CR24] Pfeiffer, T., Feiner, S. K., & Mayol-Cuevas, W. W. (2016a). *Eyewear computing for skill augmentation and task guidance (Vol. 23, p. 199)*. Schloss Dagstuhl—Leibniz-Zentrum für Informatik, Dagstuhl Publishing. 10.4230/DagRep.6.1.160.

[CR25] Pfeiffer, T., Renner, P., & Pfeiffer-Leßmann, N. (2016b). EyeSee3D 2.0: Model-based real-time analysis of mobile eye-tracking in static and dynamic three-dimensional scenes. In *Proceedings of the Ninth Biennial ACM Symposium on Eye Tracking Research & Applications*. 10.1145/2.857491.2857532 (pp. 189–196). ACM Press.

[CR26] R Core Team (2014). *R: A language and environment for statistical computing*. R Foundation for Statistical Computing. http://www.R-project.org/.

[CR27] Renner, P.,& Pfeiffer, T. (2017). Attention guiding techniques using peripheral vision and eye tracking for feedback in augmented-reality-based assistance systems. In *2017 IEEE Symposium on 3D User Interfaces (3DUI)*. 10.1109/3DUI.2017.7893338 (pp. 186–194). IEEE.

[CR28] Sidner, C. L., Kidd, C. D., Lee, C., & Lesh, N. (2004). *Where to look: A study of human–robot engagement*, (pp. 78–84). New York, NY: ACM. 10.1145/964442.964458.

[CR29] Staudte, M., Koller, A., Garoufi, K., & Crocker, M. W. (2012). Using listener gaze to augment speech generation in a virtual 3D environment. In *Proceedings of the 34th Annual Conference of the Cognitive Science Society*. Sapporo, Japan.

[CR30] Striegnitz K., Buschmeier H., Kopp S. (2012). Referring in installments: a corpus study of spoken object references in an interactive virtual environment. Proceedings of the Seventh International Natural Language Generation Conference, INLG ’12.

[CR31] Tanenhaus M. K., Spivey-Knowlton M., Eberhard K., Sedivy J. (1995). Integration of visual and linguistic information in spoken language comprehension. Science.

[CR32] Zarrieß S., Schlangen D. (2016). Easy things first: Installments improve referring expression generation for objects in photographs. Proceedings of the 54th Annual Meeting of the Association for Computational Linguistics (Volume 1: Long Papers).

